# Comparative analysis of gut and ovarian microbiota in *Dactylopius opuntiae* (Hemiptera: Dactylopiidae) from distinct Moroccan regions

**DOI:** 10.3389/fmicb.2026.1890634

**Published:** 2026-08-14

**Authors:** Oumaima Moustaid, Mohamed Idbella, Salah Eddine Azaroual, Karim El Fakhouri, Chaimae Ramdani, Mohamed Ouaarous, Mustapha El Bouhssini, Issam Meftah Kadmiri

**Affiliations:** AgroBioSciences (AgBS) Program, College of Agriculture and Environmental Sciences, Mohammed VI Polytechnic University, Ben Guerir, Morocco

**Keywords:** amplicon sequencing, *Bacillota*, *Dactylopius opuntiae*, gut microbiome, ovaries, *Pseudomonadota*

## Abstract

**Introduction:**

Investigating the hidden microbial world within insect hosts yields new insights into their biology, ecology, and adaptation. *Dactylopius opuntiae* (Cockerell), a major invasive pest of the cactus *Opuntia ficus-indica*, causes severe economic and ecological damage in Morocco and other arid regions. However, little is known about its associated bacterial microbiota and how it varies across environmental gradients. This study explored the bacterial diversity of *D. opuntiae* and evaluated how it varies across tissue types, developmental stages, and contrasting environmental regions.

**Methods:**

We characterized bacterial communities in two tissue types (gut and ovary) of adult females and in whole-body samples of nymphs, collected from two ecologically contrasting regions of Morocco: Marrakech (arid, semi-desert climate) and Al Hoceima (humid, Mediterranean climate). Using 16S ribosomal RNA (rRNA) gene sequencing, we evaluated differences in diversity, composition, and relative abundance across regions and developmental stages.

**Results:**

Sequencing generated approximately 2.84 million reads across all samples, of which 91.9% were retained after quality filtering and contaminant removal, and 91.2% were successfully classified to bacterial taxa. Our results revealed clear geographic structuring of the microbiota. In insects from Marrakech, the adult gut and ovary harboured comparatively lower bacterial diversity and were dominated by *Pseudomonadota*, whereas whole-body nymph samples were dominated by *Bacillota* (>70% relative abundance). In contrast, samples from Al Hoceima showed consistently higher bacterial richness across stages and tissues, with *Pseudomonadota* accounting for approximately 60–90% of reads. Differential abundance analysis identified several taxa with nominal tissue- and region-associated differences (*p* < 0.05), including *Thiomonas*, *Burkholderia*, and *Acinetobacter baumannii* for tissues, and *Staphylococcus xylosus*, *Klebsiella pneumoniae*, *Salmonella enterica*, and *Ferrovum myxofaciens* for regions. The most represented bacterial orders in Al Hoceima samples were *Burkholderiales*, *Enterobacterales*, *Nitrosomonadales*, and *Rhodocyclales*.

**Discussion/conclusion:**

Together, these findings highlight the spatial variability of insect–microbe association in *D. opuntiae* and point to candidate bacterial taxa whose distribution covaries with regional environmental conditions. This study provides baseline, descriptive microbiome data and a foundation for future functional investigations of microbial roles in host biology, which may in turn inform microbiome-aware pest management strategies across Moroccan agroecosystems.

## Introduction

Microbiomes are diverse, dynamic communities of microorganisms that inhabit a wide range of environments, playing essential roles in shaping the health and functioning of organisms and ecosystems (Schloter et al., 2020). The study of microbiomes has rapidly advanced in recent years due to its profound implications for both basic biological understanding and applied sciences. Insects, in particular, are the most diverse and abundant animals on the planet in terms of species richness and biomass across all ecological regions (Remmal et al., 2023). These organisms have evolved intricate interactions with their associated microbiota, which are now recognized as fundamental to their survival, development, and ecological success. Insects and microbes have formed several symbiotic interactions, which have contributed significantly to their diversity and evolution (Dubey and Rapalli, 2024). Microorganisms contribute to vital functions in insects such as detoxification, development and physiology, and immunity (Brentassi and De La Fuente, 2024).

Among the economically important host plants for such insect-microbe interactions is *Opuntia ficus-indica*, a cactus species extensively cultivated in Morocco. Introduced to the country in 1770, its cultivation has expanded from 50,000 ha in 1998 to over 150,000 ha today, with more than 40% concentrated in southwestern Morocco (MAPMDREF, 2017). This cactus plays a crucial role in local agriculture, with recognized value in the pharmaceutical and cosmetics sectors (Shetty et al., 2012). However, since 2014, cactus plantations in Morocco have been severely threatened by the invasive sap-sucking insect *Dactylopius opuntiae* (Hemiptera: Dactylopiidae) (El Fakhouri et al., 2023). Adult females, covered in a white waxy layer, are particularly destructive and pose a major challenge to pest control efforts (Mazzeo et al., 2019). In response to this invasion, Morocco has made substantial progress in developing integrated pest management (IPM) strategies (Ramdani et al., 2021), including the use of resistant ecotypes and the application of registered insecticides (Naboulsi et al., 2022). Nevertheless, the limitations of chemical control, such as the risk of resistance development, environmental contamination, and effects on non-target organisms, underscore the urgency for sustainable and biologically based alternatives, including plant extracts, crop diversification, and entomopathogenic microbes (Ramdani et al., 2021; El Fakhouri et al., 2023; Makhlouf et al., 2024).

Advances in high-throughput 16S ribosomal RNA (rRNA) gene sequencing now allow host-associated microbial communities to be characterized in detail and linked to host physiology across diverse systems. Such approaches have shown how dietary substrates are fermented by the gut microbiota and reshape community structure (Li et al., 2022), how microbiota-targeted interventions can modulate host metabolism and immunity (Su et al., 2023), and how integrating 16S rRNA sequencing with multi-omics can reveal microbiota–metabolite interactions relevant to host health (Cheng et al., 2022). As inferences drawn from amplicon data are sensitive to upstream bioinformatic choices, rigorous sequence quality control and data preprocessing are essential for reliable community profiling (He et al., 2020). Extending these approaches to *D. opuntiae* provides a route to understand how its microbiota may influence host biology and, ultimately, to inform microbiome-aware and biologically based management of this pest.

Despite the ecological and agricultural importance of *D. opuntiae*, its associated bacterial communities remain poorly characterized, and the tissue- and stage-specific structure of these communities is essentially unknown. In particular, no study has yet compared the gut and ovarian microbiota of Moroccan populations or examined how these communities vary across contrasting climatic regions. Characterizing the ovarian microbiota is especially relevant because reproductive tissues can harbour vertically transmitted symbionts that contribute to host nutrition. In Dactylopius, the nitrogen-fixing symbiont *Candidatus Dactylopiibacterium carminicum* has been reported from ovaries and hemolymph, making the ovary a logical target for symbiont surveys. To address these gaps, we characterized the bacterial communities of *D. opuntiae* at two developmental stages (nymph and adult females) and, in females, in two functionally critical tissues, the gut (nutrient absorption and metabolism) and the ovaries (reproduction and potential vertical transmission), sampled from two ecologically contrasting Moroccan regions. We tested three hypotheses: (i) gut and ovarian microbiomes differ in composition, (ii) regional environmental conditions are associated with differences in bacterial community structure, and (iii) the ovaries harbour taxa potentially involved in vertical transmission. By combining full-length 16S rRNA gene sequencing with bioinformatic profiling, this descriptive study provides a baseline for the microbial ecology of *D. opuntiae* and its potential implications for sustainable management.

## Materials and methods

### Insect sampling

As part of the microbiome investigation of *D. opuntiae*, a large number of insects were collected to provide proper representation for analysis. Specimens were primarily collected from infested cactus cladodes, with a focus on adult females covered in wax, a distinguishing feature of this species. To avoid harming the insects, soft brushes were utilized as the primary gathering tool. To allow for a thorough examination of microbial diversity, the collected individuals were carefully handled to avoid external contamination prior to laboratory preparation activities such as rinsing, dissection, and DNA extraction. The collections were carried out in two distinct regions of Morocco: Marrakech, located in the central-western part of the country, and Al Hoceima, situated along the northern Mediterranean coast. Each region was represented by three distinct places that were geographically separated to allow for varying environmental conditions (Table 1). The specimens were collected and transferred alive to the University Mohammed VI Polytechnic (UM6P) Entomology Laboratory, Morocco, where they were maintained at a constant temperature of 24 ± 2 °C, 75% humidity, and a 14:10 (light: dark) photoperiod.

**Table 1:** Sampling locations, coordinates, and associated environmental variables for *Dactylopius opuntiae* collected in Marrakech and Al Hoceima regions. Values represent annual averages of temperature, humidity, and precipitation.

Regions	Location	Samples	Coordinates		Total precipitation (mm year^−1^)	Relative humidity(%)	Temperature (°C)
			Latitude	Longitude			
Marrakech	The experimental farm UM6P	Nymph	32.2418° N	−7.9579° W	140	60.0	20.3
		Gut-Marrakech					
		Ovary-Marrakech					
	Nzalt AIT Hussain	Nymph	31.7461° N	−8.1042° W	150	54.5	21.1
		Gut-Marrakech					
		Ovary-Marrakech					
	Garden of faculty of sciences semlalia of marrakech (FSSM)	Nymph	31.6341° N	−8.0137° W	176	52.8	21.1
		Gut-Marrakech					
		Ovary-Marrakech					
Al Hoceima	Ait Hicham	Nymph	35.16122° N	−3.88189° W	298	80.2	18.7
		Gut-Al Hoceima					
		Ovary-Al Hoceima					
	Tamassint	Nymph	35.05475° N	−3.93994° W	347	61.5	17.5
		Gut-Al Hoceima					
		Ovary-Al Hoceima					
	Tala Youssef	Nymph	35.24117° N	−3.96714° W	298	80.2	18.7
		Gut-Al Hoceima					
		Ovary-Al Hoceima					

Region-level climatic descriptors were obtained from the ECMWF Reanalysis v5 (ERA5) hourly reanalysis (ERA5 single levels; Copernicus Climate Change Service, https://cds.climate.copernicus.eu) for the calendar year 2024. For each sampling site, hourly 2 m air temperature, 2 m dew-point temperature, and total precipitation were extracted in Python (xarray) for the 0.25° grid cell nearest the site Global Positioning System (GPS) coordinates. Mean annual temperature was computed as the hourly mean; mean annual relative humidity was derived from the hourly 2 m air and dew-point temperatures using the Bolton (1980) saturation-vapour-pressure formulation; and annual total precipitation was obtained by summing the hourly values. As the two northern sites, Aït Hicham and Tala Youssef, fall within the same ERA5 grid cell, they share identical climatic estimates (Table 1).

### Tissue collection

Two developmental stages were sampled: whole-body nymphs and adult females. For adult females, two tissue types (gut and ovary) were analysed, whereas nymphs were processed as whole bodies. For each region, samples were obtained from three independent locations, which served as biological replicates (Table 1). Each replicate consisted of tissue pooled from multiple individuals to obtain approximately 200 mg of starting material per sample type (gut, ovary, and whole nymph); pooling was standardized by mass because individual body size varied. Three technical replicates were processed for each pooled sample, yielding three biological replicates per tissue, stage, and region.

To preserve their genetic integrity, the nymphs were maintained in Eppendorf tubes filled with a 70% ethanol solution and stored at −20 °C. To ensure the purity and quality of the DNA isolated from *D. opuntiae* nymphs, several rigorous steps were undertaken. The nymphs’ surfaces were first sterilized by submerging them in 70% ethanol, then rinsed three times with sterile distilled water. This method removes any external contaminants that might affect the purity of the extracted DNA.

**Figure 1:**
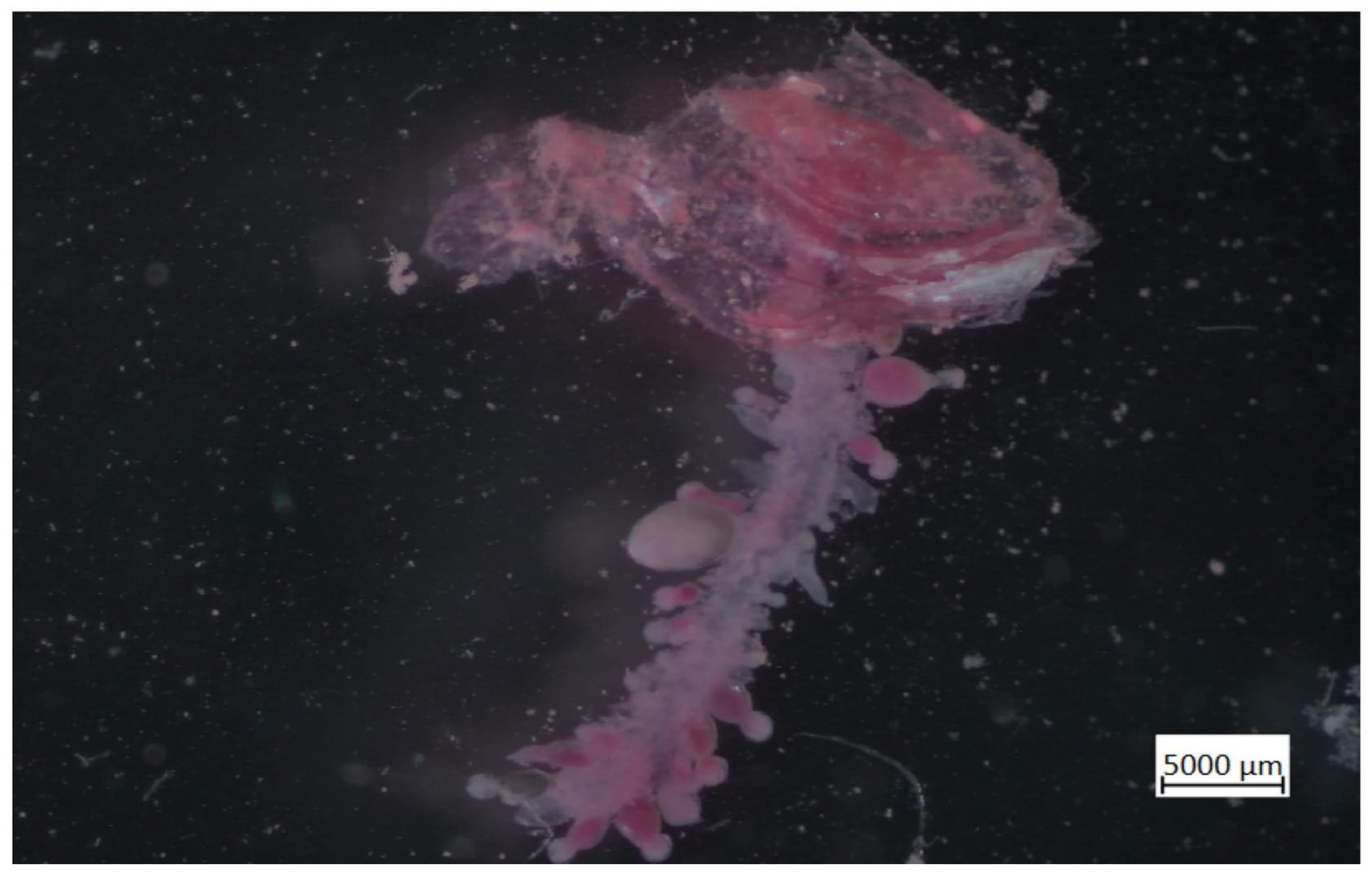
*Dactylopius opuntiae* ovary image of adult females observed under a stereoscopic microscope X5.

**Figure 2:**
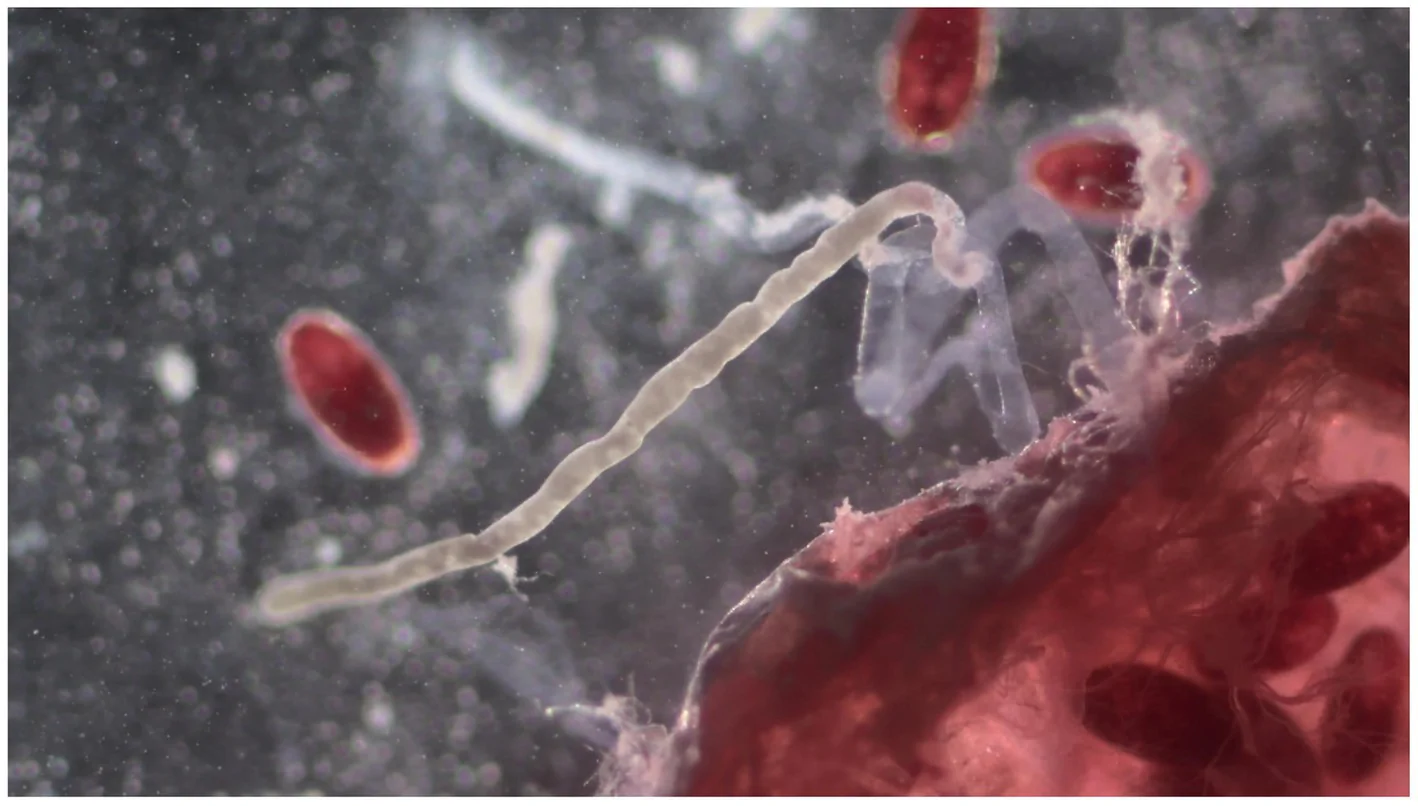
*Dactylopius opuntiae* digestive system image of an adult female observed under a stereoscopic microscope X5.

Prior to dissection, the external surfaces of adult females were cleansed using 70% ethanol. Dissections were performed using sterile forceps under a stereomicroscope to remove internal organs (ovary and gut) (Figures 1, 2). The tissues were stored at −20 °C; forceps were washed with water and ethanol after each dissection. This strict process for collecting and storing tissues preserves their morphological and molecular integrity, resulting in high-quality research data for a better understanding of cochineal biology and ecology in various Moroccan locations.

### Microbial DNA extraction

Genomic DNA was extracted with the DNeasy PowerSoil Pro kit (Qiagen, Hilden, Germany) following the manufacturer’s instructions, starting from approximately 200 mg of pooled tissue per sample. To monitor background contamination, extraction blanks (no-template controls) were processed alongside the samples and carried through library preparation and sequencing as negative controls. DNA concentration and purity were assessed using a Qubit fluorometer (Invitrogen, Waltham, MA, USA) and a NanoDrop spectrophotometer (A260/A280 ratio). All DNA samples were stored at −20 °C until polymerase chain reaction (PCR) amplification and sequencing.

### PCR amplification, library preparation, and ONT sequencing

#### PCR amplification

The near-full-length 16S rRNA gene (V1–V9) was amplified using the 27F/1492R primer (Oxford Nanopore Technologies, Oxford, UK) set supplied with the Oxford Nanopore Technologies (ONT) (Oxford, UK) 16S Barcoding Kit (SQK-RAB204); this kit-specific chemistry employs LongAmp Taq (New England Biolabs (NEB), Ipswich, MA, USA), which is optimized for the long (~1.5 kb) amplicons generated by full-length 16S barcoding. Each 50 μL reaction contained 14 μL nuclease-free water, 10 μL input DNA (10 ng), 1 μL barcoded 16S primer (10 μM), and 25 μL LongAmp Taq 2X Master Mix (New England Biolabs (NEB), Ipswich, MA, USA). Thermocycling comprised an initial denaturation at 95 °C for 1 min; 25 cycles of 95 °C for 20 s, 55 °C for 30 s, and 65 °C for 2 min; a final extension at 65 °C for 5 min; and a hold at 4 °C. Negative (no-template) controls were amplified in parallel.

#### Libraries preparation

Each DNA sample (≥1 ng·μL^−1^) was used to create the 16S rRNA gene sequencing library using Oxford Nanopore Technologies’ (ONT) 16S Barcoding kit (SQK-RAB204). Agencourt AMPure XP beads (Beckman Coulter, USA) were used to purify PCR products in accordance with the manufacturer’s instructions. Each sample combination was then placed on a base magnet for ethanol washing (freshly made 70%) after being incubated in a HulaMixer (Waltham, MA, USA) for 5 min at room temperature. The pure product was eluted in 10 μL of elution buffer (10 mM Tris–HCl pH 8.0, 50 mM NaCl) after the washing stage with magnetic beads.

Each library’s concentration was measured using a Qubit dsDNA HS Assay kit (Life Technologies, UK). Following the manufacturer’s instructions, pooled libraries were prepared for the sequencing phase and placed into a MinION flow cell (R9.4.1, Flo-Min106D). MinKnow software version 21.11.8 was used to monitor the flow cell once it was inserted into the MinION sequencer (Oxford Nanopore Technologies).

### Bioinformatic and statistical analyses

Basecalled FASTQ files generated by Guppy version 6.4.6 (Oxford Nanopore Technologies) were subjected to quality filtering using NanoFilt version 2.8.0 with the following thresholds: minimum read length = 500 bp, minimum average read quality score = Q7 (corresponding to 80% accuracy). Reads shorter than 500 bp or with average quality below Q7 were discarded. Adapter sequences were removed using Porechop version 0.2.4 with default parameters (--discard_middle, --min_trim_size 4). Demultiplexing was performed based on barcode sequences provided in the ONT 16S Barcoding Kit (SQK-RAB204).

Sequencing generated approximately 2.84 million raw reads across all samples. After quality filtering, adapter trimming, and contaminant removal, 2.61 million reads (91.9%) were retained for downstream analyses. Kraken2 successfully assigned 2.38 million reads (91.2% of filtered reads) to bacterial taxa, while 8.8% remained unclassified. Sequencing depth ranged approximately 48,000–176,000 reads per sample, with a mean depth of 132,000 reads per sample.

Taxonomic assignment of filtered 16S rRNA reads was performed using Kraken2 version 2.1.2 with the following parameters: --confidence 0.5 (minimum confidence score for assignment), --minimum-hit-groups 3 (minimum number of k-mers for a valid match), and --report-minimizer-data. Classification was run with 8 threads (--threads 8). The Kraken2 database used was a custom-built version (downloaded in September 2023) comprising complete bacterial and archaeal reference genomes from National Center for Biotechnology Information (NCBI) Reference Sequence (RefSeq, release 220). The database contained 21,348 bacterial and 578 archaeal genomes at the time of construction. Kraken2 was selected because full-length 16S rRNA gene sequences generated by Oxford Nanopore Technologies provide longer taxonomic signatures than short-read amplicons, improving classification accuracy when matched against comprehensive reference genome databases. The use of a custom RefSeq database further enhanced taxonomic resolution for bacterial community profiling. Although full-length 16S rRNA gene sequences provide greater taxonomic resolution than short-read amplicons, species-level assignments from 16S rRNA data remain putative, as closely related bacterial species may share nearly identical 16S rRNA gene sequences. Therefore, species-level reports throughout this manuscript should be interpreted as tentative classifications rather than definitive identifications.

To remove potential non-biological and environmental contaminants, we applied a multi-step filtering strategy. First, reads classified as “Eukaryota” (including insect host DNA) or “unclassified” were excluded from downstream analyses. Second, we performed *in silico* contaminant removal using the decontam package version 1.20 in R, applying the prevalence method (threshold = 0.5), which identifies taxa that appear more frequently in low-concentration samples as potential kit or reagent contaminants. Third, any taxon comprising less than 0.01% of total reads across all samples was considered background noise and removed. Finally, negative control samples (DNA extraction blanks and PCR blanks) were included in the sequencing run; any Amplicon Sequence Variant (ASV) or taxon present in negative controls with relative abundance ≥0.1% of that in biological samples was removed from all samples. A summary of the filtering steps is provided in Supplementary Table S1.

Following contaminant removal, Kraken2 report files were parsed using R version 4.3.1 with the tidyverse package version 2.0.0. A total of 3.7% of reads were removed during contaminant filtering, including reads identified by the decontam package and taxa detected in negative controls. Relative abundance values were normalized by the total number of classified reads per sample. Rarefaction was not applied because sequencing depth was comparable among samples and relative abundance normalization was used for downstream analyses. Alpha diversity metrics were calculated from the normalized abundance tables, while Bray-Curtis dissimilarities were used to assess beta diversity patterns. Missing hierarchical classifications were filled in within each sample using the fill_taxonomy function from the microbiome package version 1.22.0. All taxa names were standardized to remove formatting inconsistencies (e.g., trailing underscores and prefixes). A consolidated abundance matrix was generated at phylum, class, order, family, genus, and species levels for downstream statistical and ecological analyses.

To evaluate whether sequencing depth was sufficient to capture bacterial diversity, rarefaction curves (observed species vs. number of reads) were generated for each sample using the rarefy function from the vegan package version 2.6–4 in R (Supplementary Figure S1). Rarefaction was not applied to normalize sequencing depth prior to downstream analyses because all samples reached a plateau in rarefaction curves, indicating adequate depth to capture the majority of bacterial diversity, and also because relative abundance normalization was used for diversity and composition analyses, which is appropriate when sequencing depths are comparable and coverage is high.

Alpha diversity metrics, including the number of individuals, species richness, and Shannon diversity index, were computed to evaluate microbial richness and evenness across samples. Taxonomic composition was visualized through stacked bar plots at both the phylum and order levels, allowing for comparative profiling of dominant taxa. A heatmap of relative abundances at the species (or lowest resolved taxonomic) level was generated using Plymouth Routines in Multivariate Ecological Research (PRIMER) version 7 to visualize fine-scale compositional differences among samples. Differences in bacterial community composition among tissues, developmental stages, and geographic regions were explored using non-metric multidimensional scaling (NMDS) based on Bray-Curtis dissimilarities. NMDS was used as an exploratory ordination approach to visualize patterns of community similarity among samples. Stress values were calculated to assess the quality of ordination, with values <0.1 indicating excellent representation of community dissimilarities. To identify shared and unique taxa across experimental groups, an UpSet plot was generated using presence–absence data, enabling quantification of overlapping and exclusive microbial taxa. Finally, a principal component analysis (PCA) was conducted to assess the relationships between microbial diversity and richness metrics and environmental variables, including humidity, temperature, and precipitation. PCA loadings or contribution values are presented in the Supplementary material.

To statistically identify taxa associated with tissue types and geographic regions, we performed differential abundance analysis on centred log-ratio (CLR)-transformed relative abundances using the *compositions* package in R. The centred log-ratio (CLR) transformation was applied to address the compositional nature of microbiome data, where relative abundances sum to a constant and are therefore not independent. For tissue comparisons (gut, ovary, and nymph), we used the Kruskal–Wallis (KW) test, followed by Dunn’s *post-hoc* test for pairwise comparisons when the overall test was significant. For regional comparisons (Marrakech vs. Al Hoceima), we used the Wilcoxon rank-sum test. Multiple testing correction was applied using the Benjamini-Hochberg method to control the false discovery rate (FDR) at 0.05. All statistical analyses were conducted in R version 4.3.1 using the *vegan*, *rstatix*, *compositions*, and *dplyr* packages.

Statistical comparisons of alpha diversity indices (Shannon index, species richness) between groups (tissue types, regions, developmental stages) were performed using non-parametric Kruskal–Wallis tests, followed by Dunn’s test for multiple pairwise comparisons with Benjamini–Hochberg correction. A significance threshold of *p* < 0.05 was applied. All statistical analyses were conducted in R version 4.3.1 using the vegan and rstatix packages.

## Results

This study aimed to investigate bacterial diversity and richness in the gut and ovary of adult female *D. opuntiae*, as well as in nymphs collected from two distinct regions in Morocco, Marrakech and Al Hoceima, to assess potential regional differences in microbiome composition.

**Figure 3:**
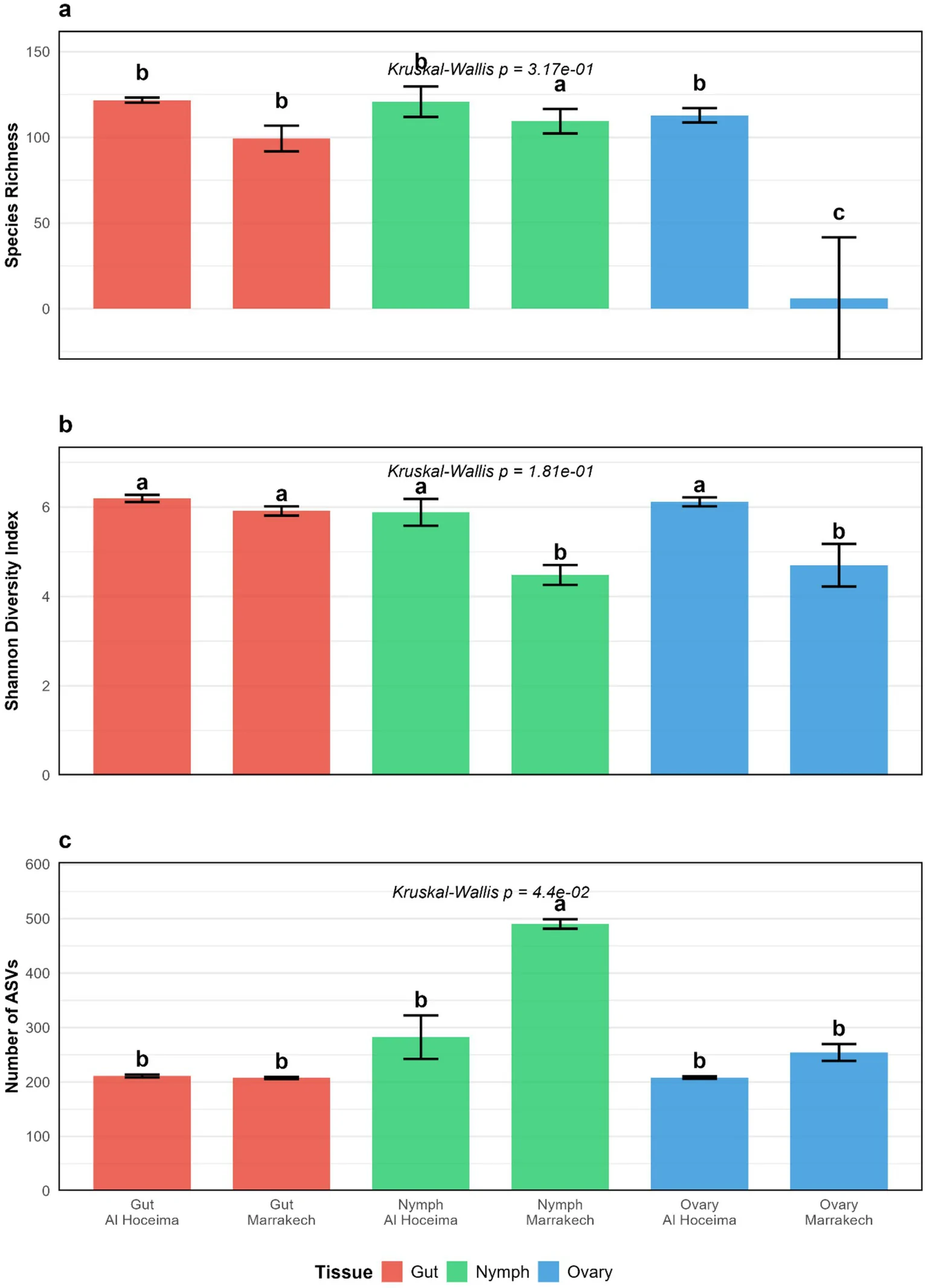
Alpha diversity of the *D. opuntiae* microbiome across different tissues (gut, nymph, and ovary) and regions (Al Hoceima and Marrakech). **(a)** Species richness, **(b)** Shannon diversity index, and **(c)** number of amplicon sequence variants (ASVs). Bars represent mean values with error bars. Different lowercase letters above the bars indicate significant differences among groups. Kruskal–Wallis p-values are shown for each diversity metric.

Alpha diversity of the cochineal microbiome across different tissues nymph, ovary, and gut in the Marrakech and Al Hoceima regions, as illustrated by species richness, individual number, and Shannon diversity index, is presented in Figure 3.

The nymph microbiome exhibited the highest species richness, followed by the ovary and gut microbiomes. Species richness was highest in nymph samples collected from Marrakech (>400 observed taxa), whereas nymph samples from Al Hoceima contained more than 200 observed taxa. However, despite their higher richness, Marrakech nymph samples exhibited comparatively lower Shannon diversity, indicating reduced community evenness and a greater dominance of a limited number of bacterial taxa. In contrast, gut and ovary samples displayed more even distributions of taxa, resulting in higher Shannon diversity values. These findings highlighted significant regional and developmental variation.

**Figure 4:**
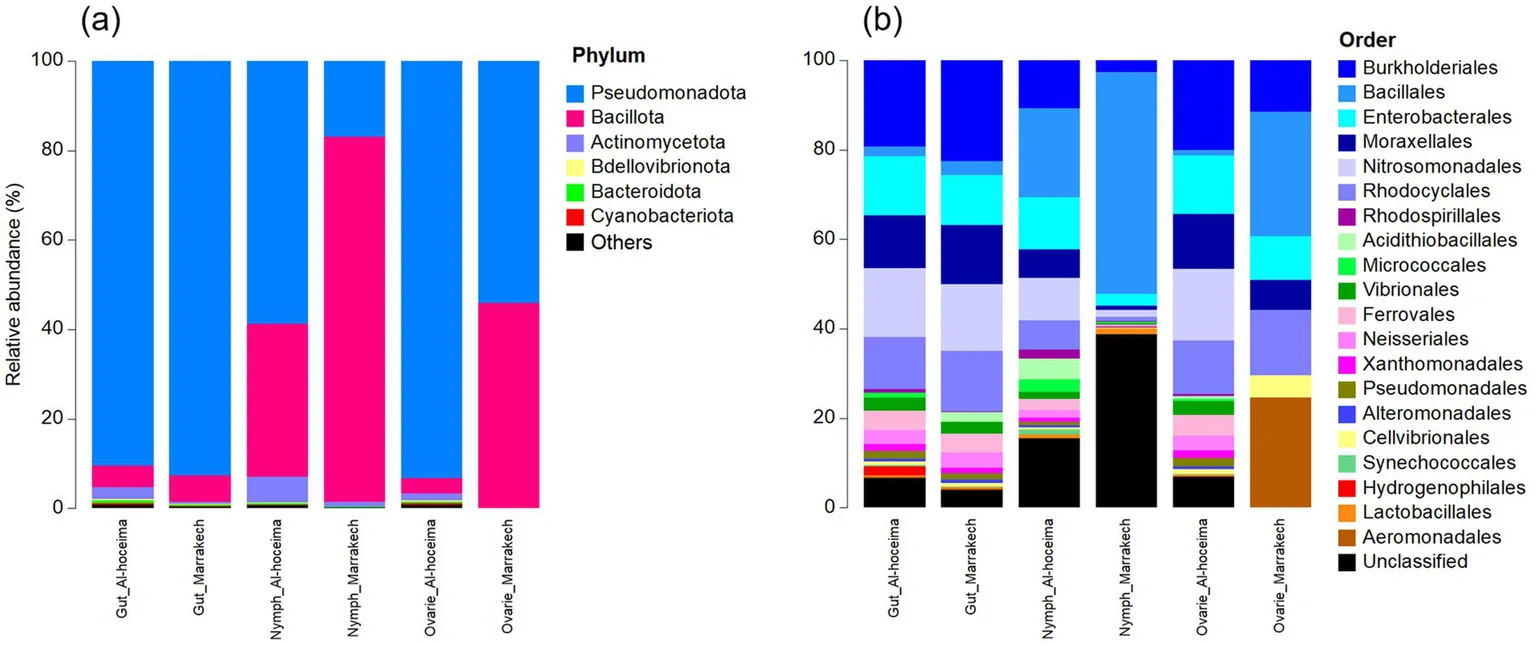
Relative abundance of the bacterial composition of *Dactylopius opuntiae* among nymphs, gut, and ovaries across Marrakech and Al Hoceima regions. **(a)** Dominant phyla, **(b)** dominant orders.

The bacterial composition showed significant differences between tissues and regions (Figure 4). In *D. opuntiae* samples from all regions, three major phyla were identified: *Pseudomonadota*, *Bacillota*, and *Actinomycetota*. *Pseudomonadota* showed *the* highest relative abundance in the gut samples from Marrakech. However, in the Al Hoceima samples, the relative abundance of *Pseudomonadota* ranged from 60 to 90% (Figure 4a), suggesting its dominance in the microbial community structure. Nymphs from the Al Hoceima region exhibited a distinct bacterial community composition, dominated by *Pseudomonadota* (>40%), followed by *Bacillota* (20–30%), whereas *Actinomycetota* contributed less than 10% to the overall relative abundance (Figure 4a). In contrast, the *Bacillota* phylum was more abundant in nymph tissues of insects collected from Marrakech areas, with more than 70%; *Actinomycetota* showed less than 1% relative abundance (Figure 4a). When comparing ovary samples from the two regions, the relative abundance of *Pseudomonadota* was higher in Al Hoceima compared to Marrakech. Furthermore, *Bacillota* and *Pseudomonadota* showed a high abundance in samples collected from the Marrakech region (Figure 4a).

**Figure 5:**
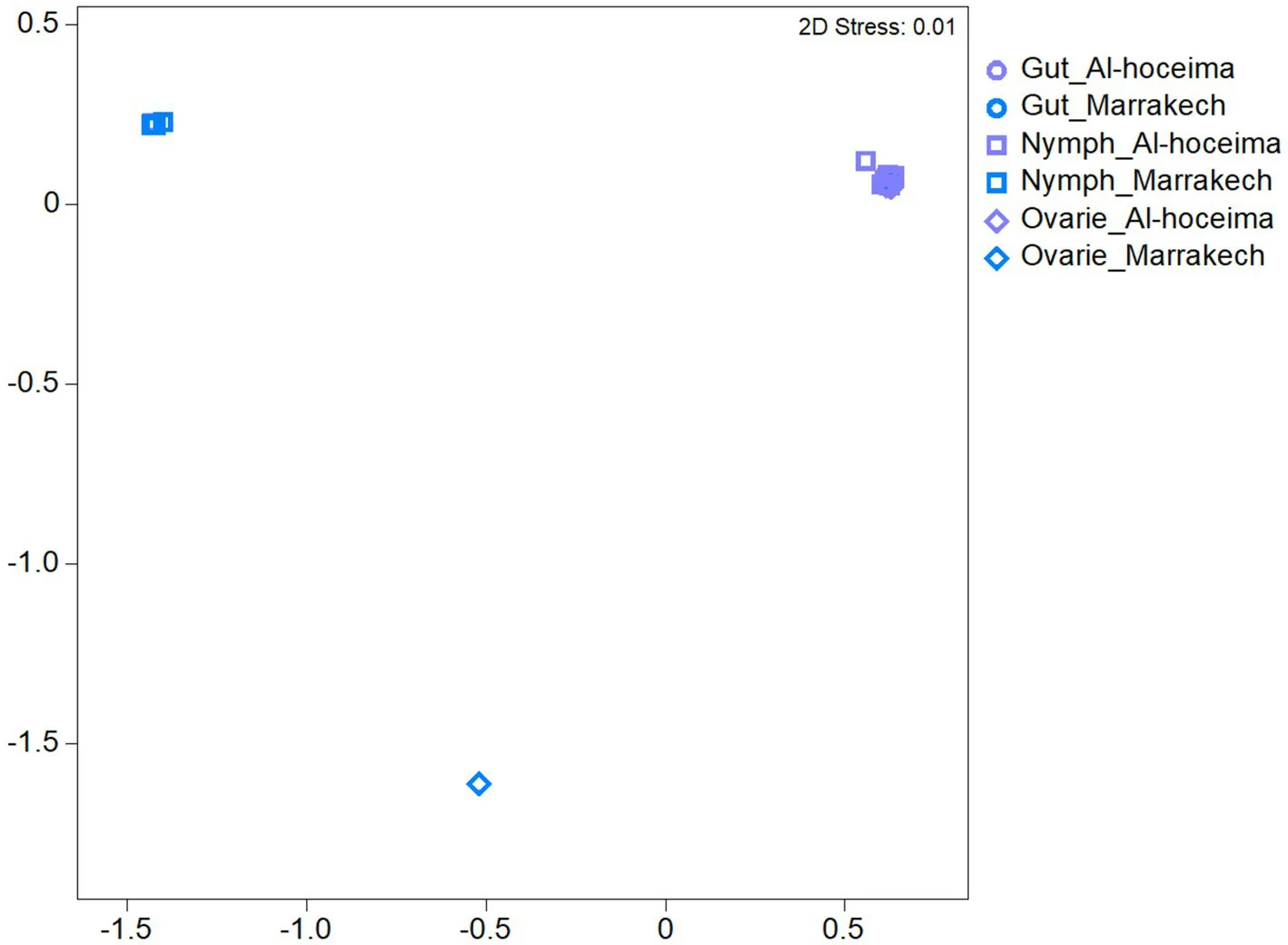
Non-metric multidimensional scaling (NMDS) of microbial communities among nymph, gut, and ovaries across Marrakech and Al Hoceima regions. Stress level shown in each plot indicates how well the individual distances between samples are represented.

NMDS ordination revealed clear differences in bacterial community composition among tissues and geographic regions (Figure 5). Samples from Al Hoceima tended to cluster separately from those collected in Marrakech, while tissue-specific patterns were also apparent. The NMDS stress value was 0.01, indicating an excellent representation of community dissimilarities in two-dimensional space. The main bacterial groups associated with Al Hoceima samples were *Burkholderiales*, *Nitrosomonadales*, and *Rhodocyclales* (Figure 4b). In contrast, samples from Marrakech displayed greater separation among tissues, suggesting bigger tissue-associated differences in community composition. The order Bacillales was particularly abundant in nymph samples collected from Marrakech. Moreover, in the ovaries of Marrakech samples, the most dominant bacterial orders were *Aeromonadales*, *Bacillales*, *Rhodocyclales*, and *Burkholderiales*, *Bacillale*s, *Rhodocyclales* and *Burkholderiales*. Moreover, *Enterobacterales*, *Moraxellales*, and *Cellvibrionales* were the least abundant in the ovaries-Marrakech. Finally, *Aeromonadales* were present only in the ovaries-Marrakech, while *Acidithiobacillales*, *Micrococcales, Vibranales*, *Ferrovales*, *Neisseriales*, and *Xanthomonadales* were completely absent (Figure 4b).

**Figure 6:**
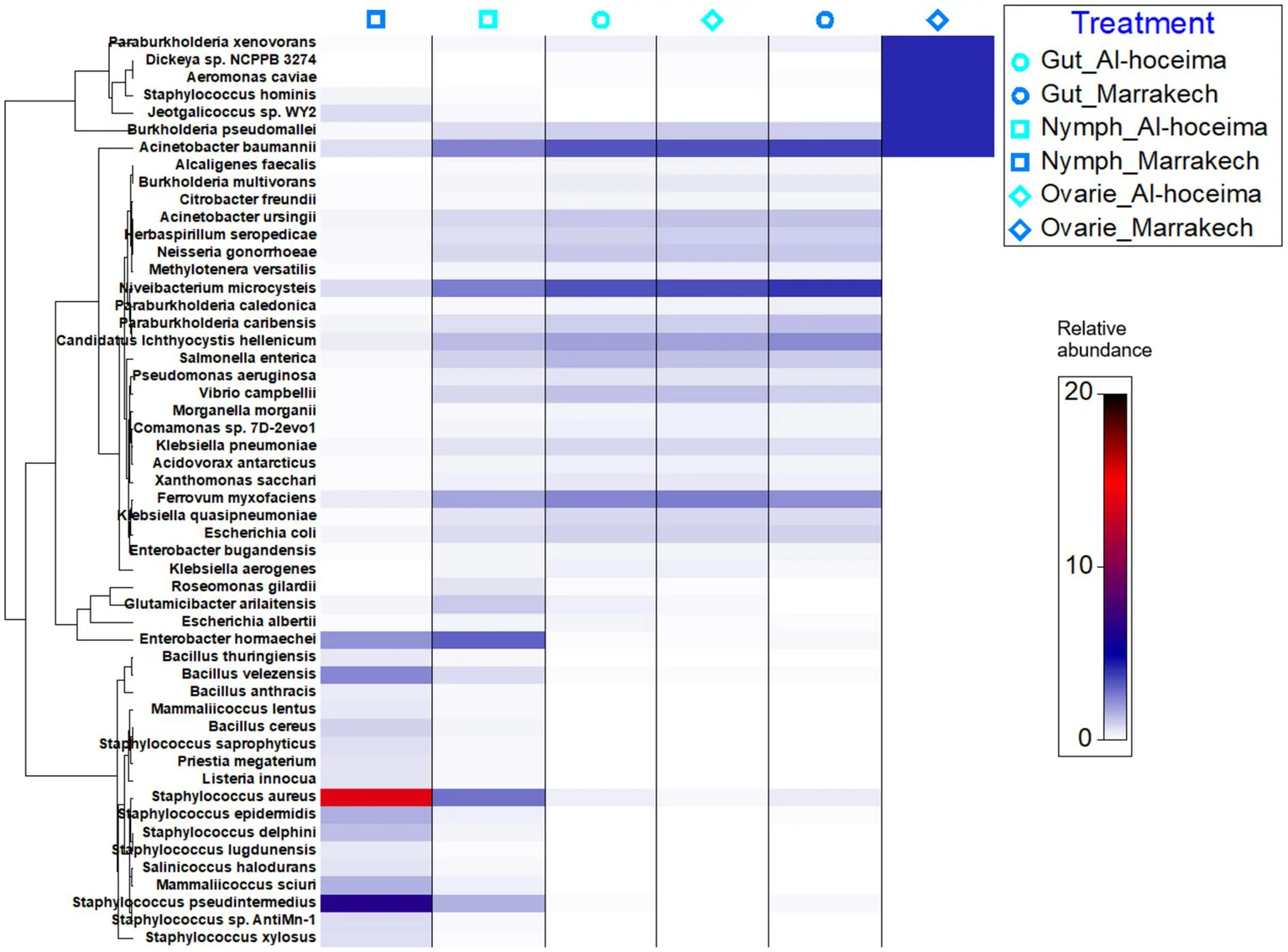
Heatmap showing the bacterial communities at the species level in the gut (blue circle), nymph (blue square) and ovaries (blue diamonds), based on 16S rRNA gene amplicon sequencing across Marrakech (dark blue) and Al Hoceima regions (light blue). Percentage relative abundance is colour coded from light blue, through red to black. The latter presents the highest abundances.

Regarding the bacterial community, a more diverse pattern was observed in the nymph samples (Figure 6). In the gut and ovaries of Al Hoceima, *Acinetobacter baumanii* and *Niveibacterium microcysteis* showed the highest relative abundance (Figure 6). Whereas *Dickeya* sp., *Aeromonas* sp., and *Jeotgalicoccus* sp., were present only in the ovaries-Marrakech. Moreover, *Paraburkholderia* sp., showed high abundance in the ovaries-Marrakech. Notably, *Enterobacter homoechei* and *Bacillus thuringiensis* were detected only in the nymph across both regions.

To statistically identify taxa associated with tissue types and geographic regions, we performed differential abundance analysis on CLR-transformed relative abundances. For the tissue comparison (gut, ovary, and nymph), the Kruskal-Wallis test revealed eight taxa with nominal differences across tissues (*p* < 0.05; Supplementary Figure S2), including *Thiomonas* (KW statistic: 6.8), *Burkholderia* (KW statistic: 7.2), *A. baumannii* (KW statistic: 7.2), and *Paraburkholderia* (KW statistic: 7.2).

**Figure 7:**
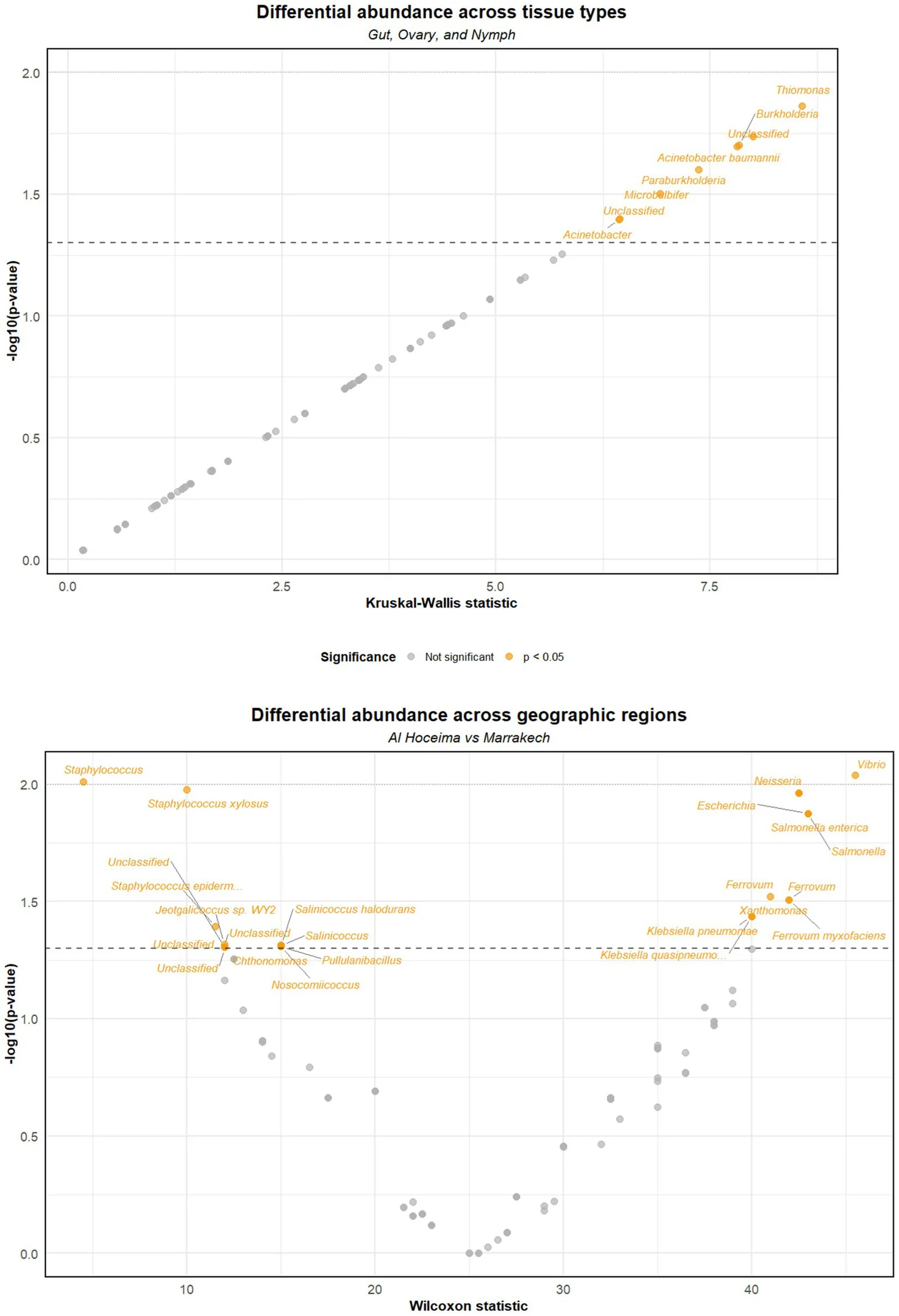
Volcano plots of differential abundance across tissue types (upper) and geographic regions (lower). Points are coloured by significance category: grey = not significant, orange = *p* < 0.05 (nominal), red = FDR < 0.05 (none observed). The horizontal dashed line indicates *p* = 0.05.

For the regional comparison (Marrakech vs. Al Hoceima), the Wilcoxon rank-sum test identified several taxa with nominal differences (*p* < 0.05; Figure 7). These included *S. xylosus*, *Jeotgalicoccus* sp. WY2, *Salinicoccus halodurans*, *Pullulanibacillus*, *Nosocomicrococcus*, *Klebsiella quasipneumoniae*, *K. pneumoniae*, *S. enterica*, *F. myxofaciens*, *Xanthomonas*, *Neisseria*, *Escherichia*, and multiple *Vibrio* species.

**Figure 8:**
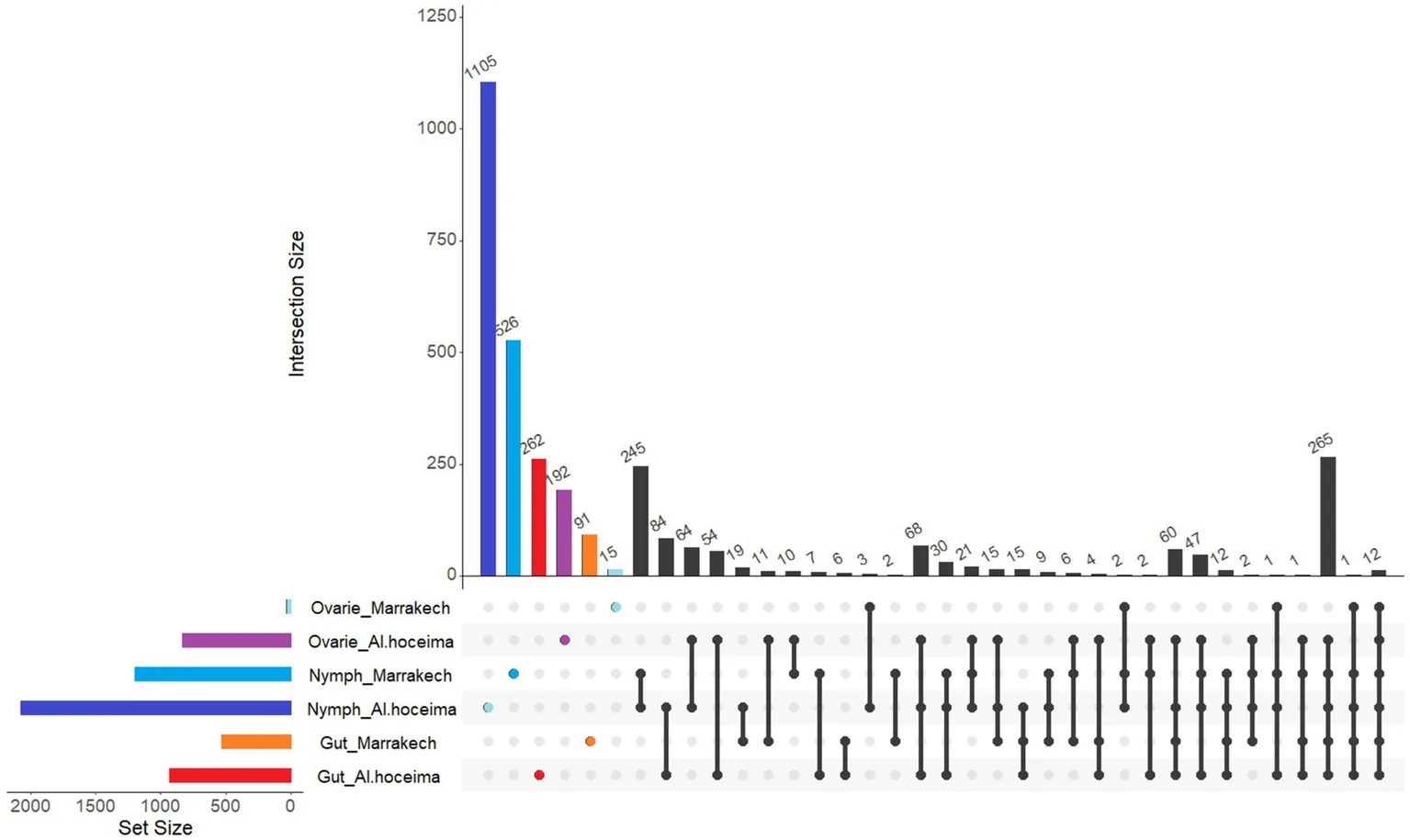
Microbiome diversity across developmental stage and tissue. UpSet analysis provides the bacterial distribution across different samples (ovaries, nymphs, and gut) from two geographical locations (Marrakech and Al Hoceima); the analysis highlights both unique and shared microbial communities.

The nymph samples showed the highest number of bacterial taxa, particularly from Al Hoceima (Figure 8), suggesting greater microbial diversity at this developmental stage. Conversely, ovary and gut samples contain fewer unique taxa, suggesting more specialized microbial communities. About 1,105 Taxa were identified in Nymph samples from Al Hoceima, followed by 526 Taxa in Marrakech (Figure 8). Furthermore, 265 taxa represent the core microbiome (Figure 8), suggesting that these core taxa may play essential roles in insect physiology and adaptation.

This UpSet analysis demonstrates that microbiome composition is influenced by developmental stage, tissue types, and geographic region.

**Figure 9:**
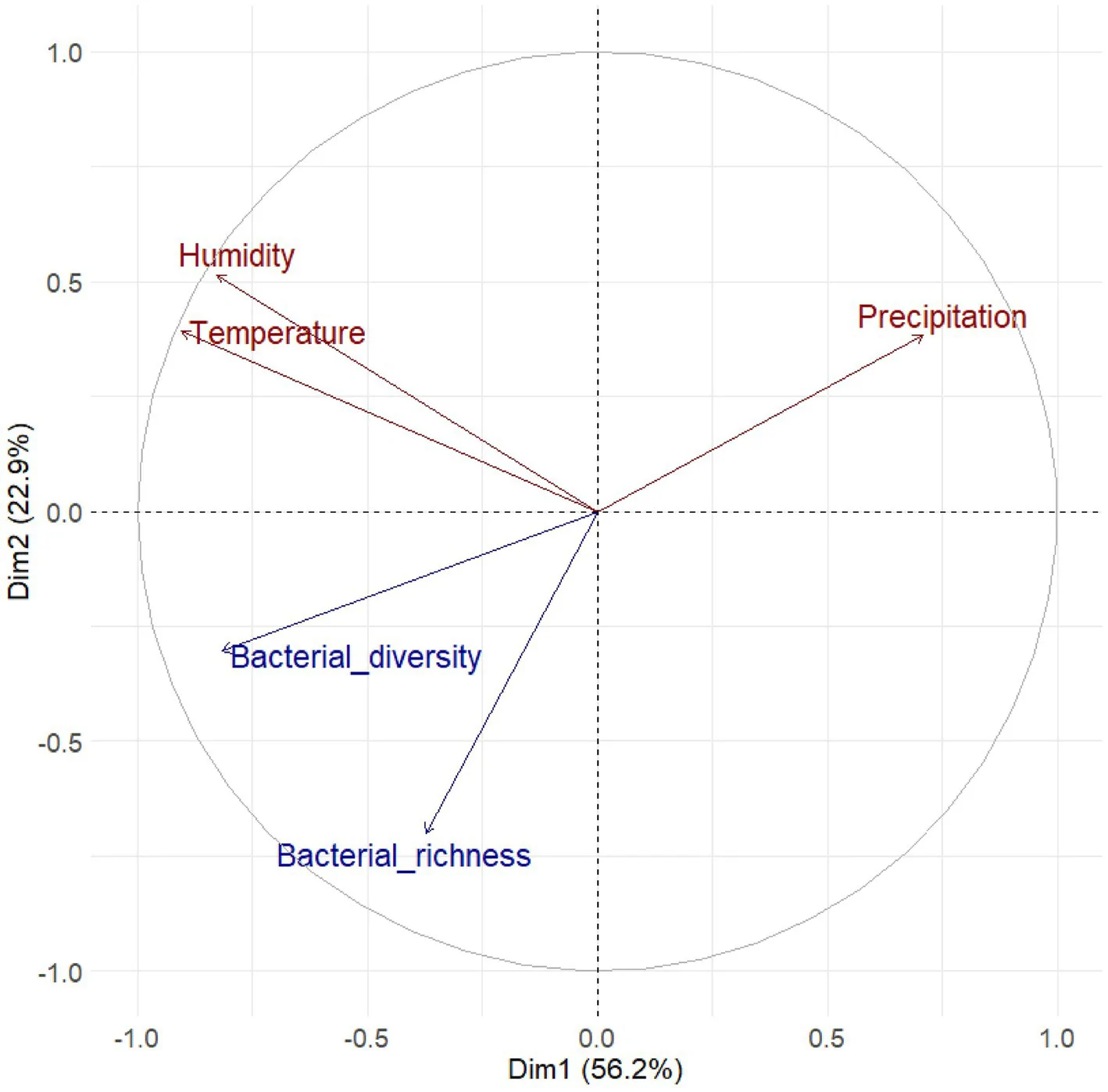
The principal component analysis (PCA) provides insight into the relationship between environmental factors (temperature, humidity, and precipitation) and bacterial community parameters (richness and diversity). The first two components (Dim = 56.2, Dim2 = 22.9%) together explain 79.1% of the total variance, indicating a strong representation of the dataset structure.

The PCA suggested an association between environmental variables (temperature, humidity, and precipitation) and bacterial community parameters (richness and diversity) (Figure 9). Samples characterized by higher values of climatic variables tended to be positioned opposite to richness and diversity vectors along the first principal component, indicating a potential inverse relationship. Precipitation exhibited the strongest contribution to PC1 (56.2% of explained variance), while temperature and humidity showed similar loading patterns, suggesting that these variables may be partially correlated. As PCA is an exploratory ordination approach, these relationships should be interpreted as associations rather than evidence of direct causal effects. These results highlight the important role of climatic variables in structuring microbial communities. The future studies incorporating a larger number of sites and formal regression-based approaches will be necessary to disentangle the relative contribution of individual environmental drivers.

## Discussion

This study investigated the presence and abundance of various bacterial taxa across different tissues of *D. opuntiae* collected from multiple regions in Morocco (Guizzo et al., 2020), providing insight into tissue-specific microbial composition and potential ecological adaptation.

It should be emphasized at the outset that the functional roles discussed, including nitrogen fixation, detoxification, antimicrobial production, vertical transmission, and host adaptation, were not experimentally tested in the present study. Our data are based on 16S rRNA gene amplicon profiling, which characterizes community composition rather than function; all such roles are inferred from previous literature on related taxa and are presented as hypotheses and potential ecological implications to be verified by the future functional, metagenomic, or culture-based work, rather than as conclusions drawn from the current dataset. We also acknowledge that the taxonomic assignments presented here, particularly at the species level, are based solely on 16S rRNA gene sequences. While the use of full-length Nanopore sequencing improves resolution compared to short-read approaches, species-level identifications from 16S rRNA data remain tentative. The taxa reported should therefore be interpreted as putative assignments.

We identified *Bacillota* as an abundant phylum across sample types in both regions. Several *Bacillus* and related taxa act as insect symbionts and have been associated with antimicrobial activity in diverse insects (Akbar et al., 2018). The dominance of *Bacillota* in our samples may therefore be consistent with a functional role in host defence and microbial competition; however, because no functional, metagenomic, or culture-based analyses were performed in this study, such roles remain hypotheses to be tested experimentally. As their symbiotic interactions with various microbes, insects can be found in a wide range of settings on Earth. Evolutionary (natural selection, mutations, gene flow, and genetic drift) and ecological (environmental aspects impacting living things) forces have led to the establishment of insect microbiota comprising representatives from different microbial domains across various species. Insect symbionts produce metabolites during their life cycle as part of their defence and feeding processes. However, the gut microbiota of bugs is a promising source of enzymes, antibacterial chemicals, and probiotics for biotechnological applications (Akbar et al., 2018). Furthermore, bacteria associated with insects belong to the families *Bacillaceae*, *Pseudomonadaceae,* and *Staphylococcaceae* and contribute to their host’s nutrition by creating chemicals and enzymes that may degrade natural and synthetic organic molecules into soluble forms, thereby boosting digestive processes (Almeida et al., 2017). These functions have been described in other insects and lead us to hypothesize that the taxa detected here could similarly contribute to nutrient acquisition and metabolic adaptation of *D. opuntiae.*

This study found different bacterial species associated with *D. opuntiae*, some of which are classified as potential pathogens, such as *Staphylococcus*, which was identified in different tissue types. This is confirmed by findings on the horn fly, in which *Staphylococcus* species such as *S. aureus* and *S. saprophyticus* have similarly been reported (Palavesam, 2012). The presence of such bacteria may reflect opportunistic associations or transient microbiota influenced by environmental exposure, rather than strictly pathogenic interactions.

Detoxifying functions are frequently attributed to gut bacteria and have been proposed to be important in several predators of *D. opuntiae*, such as *Laetilia coccidivora* and the specialist predators *Leucopis bellula* and *Hyperaspis trifurcata*. Gut-associated bacteria in these predators may metabolize carminic acid; for example, *L. coccidivora* larvae carry *Bacillus cereus*, *Enterococcus gallinarum*, and *Enterococcus casseliflavus*, *H. trifurcata* larvae carry *Enterobacter* species, and adults carry *Lactococcus lactis* and *Staphylococcus* species (Martínez-Martínez, 2023). By analogy, similar microbially mediated detoxification could occur in *D. opuntiae*; however, this remains speculative, as no detoxification assays or functional analyses were performed in the present study.

In contrast, characterization studies of the bacterial microbiome in carmine cochineal insects have highlighted the presence of *Betaproteobacteria* and the *Rhodocyclaceae* family in *D. coccus* and *D. opuntiae* ovaries, with *Dactylopiibacterium* being the dominant genus (Vera-Ponce De León et al., 2017). The detection of these taxa in reproductive tissues suggests a stable and possibly vertically transmitted symbiotic relationship.

*Dactylopiibacterium’*s genomic analysis revealed its association with the *Rhodocyclaceae* family, which shares a core genome of 293 single-copy genes found in *D. coccus* and *D. opuntiae*, with intact sequences resembling those of *Burkholderia*, while other incomplete portions were connected to *Bacillus*, *Burkholderia*, and *Pseudomonas* phages (Vera-Ponce De León et al., 2017). Such genomic features have been interpreted as reflecting a complex evolutionary history and potential functional versatility within the host; however, these inferences derive from prior genomic studies and were not evaluated in the present dataset.

The microbiota of *D. opuntiae* also included *Aeromonadales* and *Nitrosomonadales,* taxa reported to have either pathogenic or symbiotic associations (Janda and Abbott, 2010) and roles in ammonia oxidation (Hampel et al., 2020). *Dactylopiibacterium* has been detected in several *Dactylopius* species, including *D. coccus*, *D. confusus*, and *D. opuntiae*, and notably in the ovaries of *D. opuntiae* and *D. coccus*, where it has been proposed to function in symbiosis and vertical transmission (Ramírez-Puebla et al., 2010; Vera-Ponce De León et al., 2017). This genus belongs to the *Rhodocyclales* (Betaproteobacteria) and is closely related to the nitrogen-fixing *Azoarcus*; nitrogen-fixing activity and nifH expression have been reported in the hemolymph and ovaries of *Dactylopius* (Vera-Ponce De León et al., 2017). These observations, obtained in other studies, suggest that nitrogen-fixing symbionts could contribute to host nutrition in nutrient-limited environments; however, nitrogen fixation and nifH expression were not assessed in the present study, and this interpretation therefore remains hypothetical.

Our differential abundance analysis identified several taxa with significant nominal differences (*p* < 0.05) between tissues and regions, including *Thiomonas*, *Burkholderia*, *A. baumannii*, and *Paraburkholderia* for tissues, and *S. xylosus*, *Staphylococcus epidermidis*, and *Jeotgalicoccus* sp. WY2 for regions. These statistically supported differences (p < 0.05) are consistent with the broader community-level patterns observed in the NMDS ordination and the alpha diversity analyses, reinforcing the biological relevance of these candidate taxa. The significant differences in *Thiomonas* and *Burkholderia* across tissues are particularly noteworthy, as these genera have been associated with sulphur oxidation and nitrogen fixation, respectively, in other insect systems (Vera-Ponce De León et al., 2017; Hampel et al., 2020). Similarly, the significant regional differences in *Staphylococcus* species and *Jeotgalicoccus* may reflect environmental influences on the insect microbiota, as these taxa are commonly found in soil and plant-associated environments and may be acquired through environmental exposure.

A further consideration concerns the scope of the sampling design. The two regions studied, Marrakech and Al Hoceima, were deliberately selected as strongly contrasting agro-climatic zones an arid, semi-desert interior and a humid, Mediterranean coastal area separated by several hundred kilometres that represent settings in which *D. opuntiae* is an established and economically damaging pest in Morocco; this marked environmental contrast, with each region sampled at three geographically separated sites, is a deliberate strength of the comparison. At the same time, because only two regions were examined and individuals were pooled within each per-site replicate, among-individual (host-associated) variability could not be quantified, and differences between the two regions cannot be fully separated from other, unmeasured factors that co-vary between these areas. The patterns reported here are therefore best regarded as robust exploratory contrasts between two key affected regions. Confirming how far this variability generalizes will require extending the survey to additional regions spanning the climatic range of the pest, together with individually processed (non-pooled) replicates, in the future study.

One important point to highlight is that there is a limitation of the present study in terms of the taxonomic assignments, which were inferred from full-length 16S rRNA gene sequences. Although Oxford Nanopore sequencing provides greater taxonomic resolution than short-read approaches, species-level assignments should be interpreted cautiously because closely related bacterial species may share highly similar 16S rRNA gene sequences. Consequently, taxa reported at the species level should be considered putative identifications. The future studies integrating isolate-based characterization, whole-genome sequencing, or complementary molecular markers will be useful for confirming species-level identities and exploring their functional roles within the *D. opuntiae* microbiome.

Our PCA results revealed a negative correlation between environmental factors (temperature, humidity, and precipitation) and bacterial richness and diversity, indicating that increases in these climatic variables tend to reduce microbial diversity. Precipitation, in particular, exerted the strongest influence on bacterial community variation, suggesting that rainfall may be a key determinant in shaping microbial assemblages. The close association between temperature and humidity further highlights their similar impact on microbial structure, where higher levels may promote the proliferation of specific bacterial taxa while suppressing others, ultimately driving community shifts. These findings are consistent with previous studies demonstrating that environmental gradients strongly regulate gut microbial composition. Koch et al. (2013) reported that geographical location and temperature significantly influenced microbial diversity in insect hosts. Similarly, Zhao et al. (2018) showed that temperature and spatial factors were key drivers of bacterial variation in honeybees. It is important to note that the limited number of sampling locations in the present study (six sites across two regions) precludes robust statistical inference regarding the relative importance or causal effects of individual climatic factors. The associations observed here should therefore be considered exploratory and hypothesis-generating rather than confirmatory. The future studies with expanded geographic coverage and formal modelling frameworks will be necessary to rigorously evaluate the environmental drivers of microbiome variation in this pest species.

However, in contrast to these studies, our results indicate a stronger contribution of precipitation, which may reflect region-specific environmental conditions in Morocco or differences in host ecology. This suggests that the relative importance of climatic factors may vary depending on the ecological context and host species.

Overall, our results, together with previous evidence, indicate that climatic variables, particularly temperature and precipitation, play a pivotal role in shaping the diversity, abundance, and structure of gut bacterial communities. Fluctuations in these factors may directly alter microbial niches or indirectly influence host physiology and diet, thereby reshaping the gut ecosystem. Understanding these patterns is crucial, as shifts in microbial diversity may have functional consequences for host health, nutrition, and adaptation to environmental change.

## Conclusion

This study provides a preliminary, descriptive characterization of the bacterial communities associated with *D. opuntiae* across two contrasting agroecological regions of Morocco. Our findings reveal geographic and tissue/stage variability in microbial diversity and composition and identify candidate bacterial taxa, including potential reproductive symbionts, that warrant further investigation. Rather than supporting immediate applications, these baseline data establish a reference for the future functional studies aimed at clarifying the roles of key taxa in the biology of *D. opuntiae*. Such knowledge could, in the longer term, inform microbiome-aware and integrated pest management strategies for sustainable cactus production. Furthermore, the future studies might focus on deeper sequencing analysis and the roles of key bacterial species in the *D. opuntiae* life cycle and physiology. By focusing on these areas, we can develop a better understanding of the ecological and evolutionary relevance of these interactions, which can eventually help us manage *D. opuntiae* populations using an integrated pest management approach for sustainable agriculture.

## Data Availability

The datasets presented in this study can be found in online repositories. The names of the repository/repositories and accession number(s) can be found at: https://www.ncbi.nlm.nih.gov/, PRJNA1330813.
